# Effects of urbanisation and a wastewater treatment plant on microplastic densities along a subtropical river system

**DOI:** 10.1007/s11356-021-13185-1

**Published:** 2021-03-08

**Authors:** Tatenda Dalu, Thabiso Banda, Thendo Mutshekwa, Linton F. Munyai, Ross N. Cuthbert

**Affiliations:** 1 Aquatic Systems Research Group, School of Biology and Environmental Sciences, University of Mpumalanga, Nelspruit, 1200 South Africa; 2 Aquatic Systems Research Group, Department of Ecology and Resource Management, University of Venda, Thohoyandou, 0950 South Africa; 3 GEOMAR, Helmholtz–Zentrum für Ozeanforschung Kiel, 24105 Kiel, Germany

**Keywords:** Aquatic environment, Fibres, Freshwater ecosystem, Microbeads, Plastic pollution, Sewage treatment

## Abstract

Global freshwaters are increasingly threatened by pollutants emanating from human activities around watersheds. Microplastic pollution is an increasing problem for rivers worldwide, potentially threatening ecological integrity, ecosystem services and human health. We present quantifications and characterisations of sediment microplastic pollution in a subtropical river system in southern Africa, and relate distributions to wastewater treatment works, abiotic variables and urban environments. We additionally apply several diversity indices to decipher how microplastic types differ across the river system seasonally. Over two thousand microplastic particles were found across five sites and three seasons in the river system, comprising microbeads of various colours and microfibres. Microplastic concentrations were highest and most diverse in the hot–wet (mean range 76.0 ± 10.0–285.5 ± 44.5 microplastic kg^−1^) season as compared to the cool–dry (16.5 ± 4.5–27.0 ± 5.0 microplastic kg^−1^) and hot–dry (13.0 ± 4.0–29.0 ± 10.0 microplastic kg^−1^) seasons, and were mostly dominated by microfibres. However, no clear patterns were found in relation to wastewater treatment operations spatially, or in relation to abiotic variables in the river system. This study therefore finds a diverse range of microplastic types widely distributed in the river system that differ across seasons. Our results provide important, novel insights into plastic pollution in an understudied area of the Global South, and point to extensive pollution from sources outside of wastewater treatment works.

**Supplementary Information:**

The online version contains supplementary material available at 10.1007/s11356-021-13185-1.

## Introduction

River ecosystems are central components of the global water cycle and critical for human health, connecting inland watersheds to the marine environment and providing drinking water for most of the global population (Koelmans et al. 2016). Rivers thus serve as conduits that connect terrestrial and aquatic realms. However, given this connection by river systems, pollutants may accrue from multiple environment types in waterways (Windsor et al. 2019). In particular, microplastic pollution in aquatic systems is an increasingly pervasive international problem, with potentially severe impacts on ecosystem functioning and human health (Windsor et al. 2019; Campanale et al. 2020). Lotic environments, in particular, have a low residency time (Soballe and Kimmel 1987) and play a vital role in sediment fluxes, which in turn influence microplastic distribution (Browne et al. 2011; Cole et al. 2011).

Plastics are polymers that are intended to be lightweight, resistant and durable for domestic and industrial use. However, poor waste and general management have resulted in plastic entering aquatic environments globally in marked abundances (Thompson et al. 2009; Dalu et al. 2019; Dahms et al. 2020). Ultimately, human activities are considered major plastic sources within aquatic and terrestrial environments (de Souza Machado et al. 2018; Nel et al. 2018; Pereao et al. 2020). The pollution of plastics is worldwide in scale, with microplastic particles detected in waters and sediments, as well as in polar areas and their fauna (Obbard 2018; Bessa et al. 2019). This is despite the remoteness of these areas and is due to intensifying trade and transport networks. Microplastic sources can be categorised as primary or secondary, with primary sources being mostly from domestic, industrial and agricultural products (Fendall and Sewell 2009; Cole et al. 2011), and secondary sources being mostly fragments and microfibres due to the breaking down of larger plastic items (Browne et al. 2011; Cole et al. 2011). Thus, microplastics are typically categorised as being particles with a diameter <5 mm (Windsor et al. 2019).

The way that microplastics are distributed within aquatic ecosystems is determined by their physicochemical properties (Cable et al., 2017). In this regard, physical forces, such as waves and tides which are involved in microplastic dispersal and transport in marine systems, are similar to those of freshwater systems (Free et al. 2014; Mbedzi et al. 2020). The nature and colour of organic matter have been identified as major limitations when conducting microplastic studies in freshwaters (Nel et al. 2018, 2019), with studies greatly relying on the use of colour and shape for microplastic detection through microscopy. Other physical variables that may influence microplastic dispersal and distribution in freshwater ecosystems include water depth, substrate type, substrate embeddedness and water flow/velocity (Simpson et al. 2005; Nel et al. 2019). An example is variability in river load (i.e. sediment flux) as a river runs from upper to lower reaches, whereby high-density particles can be deposited as the river widens and water flow reduces. However, there are presently poor quantitative bases for microplastic pollution in rivers within subtropical regions of the Global South in terms of densities and type as river characteristics change spatiotemporally.

Wastewater infrastructures across the developing world remain poorly developed and managed (Mema, 2010), and wastewater discharge from these systems is regarded as an important source of microplastic contamination in aquatic ecosystems (Fendall and Sewell 2009; Browne et al. 2011; Chang 2015). Therefore, because wastewater treatment plants are unable to remove large quantities of microplastic particles, they potentially discharge great amounts into the receiving waterways daily (Eriksen et al. 2013; Mason et al. 2016; Murphy et al. 2016). In riverine systems, these plastics can be subsequently dispersed into other inland waters and the marine environment. At sewage discharge sites, fibres which are mainly from households have been found to be the most abundant and tend to have longer residence times in freshwater ecosystems (Browne et al. 2011). However, little is known about microplastic occurrence and abundance downstream of sewage treatment works across the developing world, relative to upper river reaches. In turn, this impedes understanding of the potential ecological and human health impacts of these wastewater systems and negates their improvement to reduce pollution loads.

This study aimed to investigate the effects of urban development, and in particular wastewater treatment works, on the distribution, type and occurrence of microplastic along a subtropical river system. Specifically, we examined microplastic concentrations and characteristics at five sites upstream and downstream of a water treatment plant in a riverine system, and tested for seasonal variations in microplastic pollutants. Furthermore, we correlated microplastic pollution loads with major environmental variables from study sites, to identify potential abiotic drivers of these pollutants. Specifically, we hypothesised that (i) microplastic concentrations will be relatively low upstream of the wastewater treatment plant and higher downstream due to the inefficiency of the wastewater treatment process at removing microplastics (null hypothesis: equal concentrations across river sites); (ii) upstream areas would be proportionally dominated by microbeads, and downstream areas by microfibres (null hypothesis: same microplastic type proportions among river sites); (iii) concentrations would be highest in the hot–wet season, as sedimentary sinks of microplastics become sources owing to increasing discharge (null hypothesis: equal concentrations among seasons) (Nel et al. 2018); and (iv) microplastic concentrations would relate significantly to abiotic variables such as sediment organic matter (SOM) (null hypothesis: no effect of these variables on microplastic concentrations).

## Materials and methods

### Study area

**Fig. 1:**
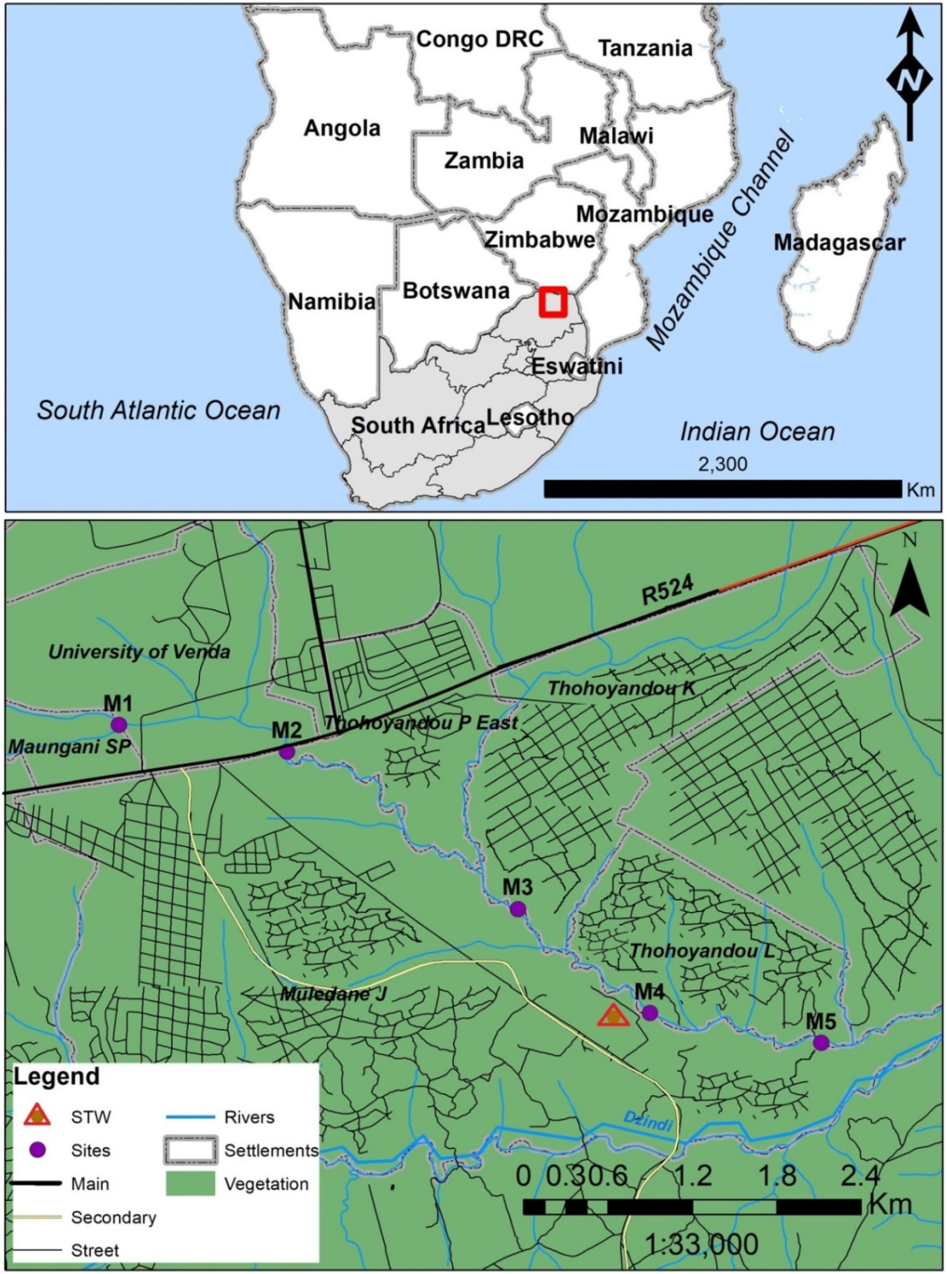
Location of the subtropical study system, Mvudi River in South Africa, highlighting the five sampling sites, wastewater treatment plant (STW) and human settlements

Sampling was conducted in five sites along an intermittent stream (class H – Hydrographic), the Mvudi River (22° 59′ 37.5″ S–23° 00′ 10.4 S; 030° 27′ 59.3″ E–030° 28′ 46.0″ E), across three seasons (i.e. cool–dry (June 2019), hot–dry (September 2019), hot–wet (February 2020)) in the Limpopo Province of South Africa (Fig. 1). Mvudi River flows through the town of Thohoyandou and into Nandoni reservoir. It is located at an elevation of 546 m above sea level. The river catchment receives high rainfall during the summer (i.e. February, ~ 284 mm) and low rainfall in winter (i.e. June, ~ 14 mm) and Spring (i.e. September, ~ 14 mm). Average temperatures for the catchment range from 20 °C (June, range: 14–24 °C) to 24 °C (February, range: 18–28 °C), with low average night temperatures of 7.5 °C occurring during July. The Mvudi River catchment is subjected to various pollution sources, such as informal and formal human settlements, water abstraction, riparian brick making, washing and bathing, subsistence and commercial agriculture, sewage discharge/spillage and solid waste disposal/dumping from nearby communities. In particular, as the river leaves the town area, effluent from Thohoyandou wastewater treatment plant is released into the river before it enters Nandoni reservoir. The reservoir supplies drinking and agricultural irrigation waters to surrounding communities and farms. Thohoyandou is located in the Thulamela Municipality, in the Limpopo Province of South Africa, with an estimated household density and population of 17,345 and 89,427, respectively (Statistics South Africa 2016). It is the administrative centre of Vhembe District Municipality and Thulamela Local Municipality. The Thohoyandou total area coverage is 42.62 km^2^ and the major economic sectors are commercial and subsistence agriculture. Informal small-scale trading is one of the most popular business ventures. Twenty-seven percent of the population are formally employed with 46% unemployed (Statistics South Africa 2016).

### Environmental variables

At each site and season, basic water parameters (i.e. conductivity (μS cm^−1^), pH, temperature (°C) and total dissolved solids (ppm)) were measured using a portable handheld multi-parameter probe (PCTestr 35, Eutech/Oakton Instruments). To determine SOM, ~3 g dry mass of homogenised sediment samples was collected, and burnt at 450 °C in a furnace for 5 h based on the loss in ignition (LOI) method (Cao et al. 2011). The SOM results were presented as percentages (%).

### Microplastic sampling and processing

Two sediment samples (~1.5–2 kg) were collected per site and season in the littoral zone (i.e. one sample was made up of three sediment subsamples collected from three random spots, approximately 10 m apart) from the upper 5 cm sediment layer along the river and stored in labelled ziplock bags. To prevent laboratory contamination, prior to all analyses, the entire laboratory was cleaned, with all surfaces and equipment cleaned with milliQ distilled water. No air-conditioners or fans were utilised in the laboratory during sample processing to minimise the potential risk of airborne microplastic particle introduction to the samples. Microplastics considered for this study were < 5 mm (GESAMP 2019), but > 63 μm (mesh size utilised). In the laboratory, the sediment samples were dried in an oven (60 °C, 72 h). After drying, each sediment sample was homogenised using a riffle splitter, and thereafter a sediment subsample of 0.5 kg was similarly separated and sieved through a 500-μm steel mesh to remove large organic matter particles and stones. The sediment material retained on the sieve was analysed under a dissecting microscope for large microplastics (500 μm−5 mm), for inclusion in the total microplastics count.

Each sieved 0.5 kg subsample was then placed into a clean 5-L beaker with a 63 μm mesh-filtered hypersaturated saline solution (100 g coarse salt L^−1^). The mixture was stirred vigorously to allow for the release and suspension of trapped plastic particles, before allowing the denser sediment to settle out for 3–24 h, depending on the soil type. After this time, the supernatant was filtered through a 63-μm mesh and the entire process was repeated five times so that all microplastics could be quantified (Nel et al. 2018). To further reduce potential airborne microplastic particle contamination, all samples were covered with a small tray. Microplastics on the 63-μm mesh sieve were carefully rinsed with distilled water into 50-mL polystyrene jars, before the samples were visually sorted under an Olympus dissecting microscope at × 50 magnification, whereby all possible microplastic particles were enumerated according to colour (i.e. red, white, blue, green for microbeads) and type (i.e. fibre or microbead). Particles were deemed to be microplastic if they possessed unnatural colouration (e.g. bright colouration, multicoloured) and/or unnatural shape (e.g. sharp edges, perfectly spherical; Hidalgo-Ruz et al. 2012). As visual inspection alone was not adequate to characterise and exhaustively quantify microplastic, further physical analysis was utilised, such as the hot needle technique (Mintenig et al. 2017; GESAMP 2019). The number of microplastic particles was estimated per kg of dry weight (dwt). Microplastic recovery rates were tested according to Mbedzi et al. (2020) and the recovery rates ranged between 86 and 97%.

### Data analysis

All data were assessed for normality and homogeneity of variance and were found to conform to parametric assumptions using the Shapiro–Wilk’s W and Levene’s tests, respectively. A two-way ANOVA (where we report F- and *p*-values) was used to analyse the differences in environmental variables, total microplastic ‘species’ (i.e. different particle types and colours; see below), as well as diversity indices among sites (M1–M5) and seasons (cool–dry, hot–dry, hot–wet), with significant variables being further assessed for pairwise differences using Tukey’s post hoc analysis (where we report *p*-values alone). All statistical analyses were carried out in SPSS version 16.0 (SPSS Inc. 2007).

Diversity analysis was calculated based on the modified Battisti et al. (2017, 2018) and Dalu et al. (2019) equations for macroplastics. The total number of microplastic particle/colour ‘species’ in each sample (a measure of γ-diversity (gamma diversity); Magurran 2004) was calculated for each site across seasons. A measure of microplastic particle/colour ‘species’ turnover inside each site per season (i.e. the Whittaker β-diversity (beta diversity)), corresponding to the internal heterogeneity in a ‘community’ or in a site, was then calculated as βW = γ/mean α (Koleff et al. 2003). Further, the Shannon–Wiener diversity index (*H′*) (Shannon and Weaver 1949) is a non–parametric diversity measure based on the number of species and relative numbers of times the species occur, and it was calculated as follows:

$$ {H}^{\prime }=-\sum fr\times \ln (fr) $$

where *fr* is the relative frequency of each microplastic particle/colour ‘species’.

The evenness index (*E*) (Wilson 1991) is a metric used for the measurement of sample heterogeneity in an assemblage based on the relative frequency distribution of species (for this study, microplastic particle/colour types). When microplastic particles/colour group evenness is high, the dominance of a specific microplastic particles/colour group becomes lower. Evenness index (*E*) was thus calculated as:

$$ E={\mathrm{H}}^{\prime }/\mathrm{H}{\prime}_{max} $$

where *H′*_max_ = lnS.

Last, Pearson’s correlations were carried out to assess the relationships between microplastic particle numbers and environmental variables. All statistical analyses were carried out in SPSS version 16.0 (SPSS Inc., 2007).

## Results

### Environmental variables

**Fig. 2:**
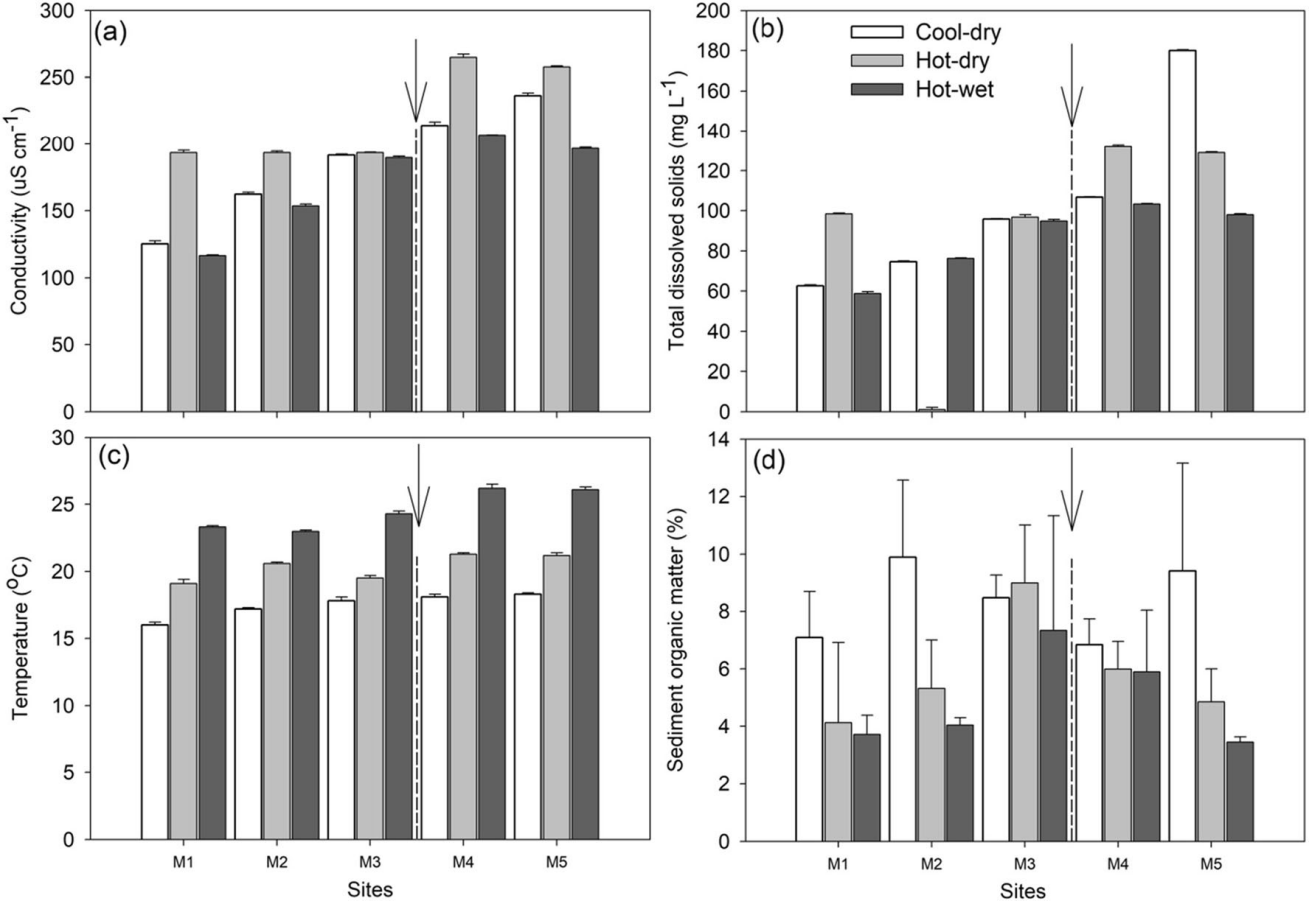
Mean (±standard error) environmental variables: (a) conductivity, (b) total dissolved solids, (c) water temperature and (d) sediment organic matter measured across three seasons and five sites in Mvudi River, South Africa. Arrows represent the position of sewage treatment works

Conductivity, total dissolved solids (TDS) and water temperature generally showed an increasing trend downstream (i.e. M1–M5) across all three seasons, and were particularly elevated downstream of the wastewater treatment plant (Fig. 2a–c). Significant seasonal differences were observed for conductivity (F = 11.07, *p* = 0.005) and water temperature (F = 46.27, *p* < 0.001), whilst significant site differences were observed for conductivity (F = 14.79, *p* = 0.001), TDS (F = 3.44, *p* = 0.037) and temperature (F = 7.97, *p* = 0.007). Using pairwise comparisons, significant differences were observed for cool–dry vs hot–dry (*p* = 0.006), cool–dry vs hot–wet (*p* < 0.001) and hot–dry vs hot–wet (*p* = 0.006) for temperature, with no significant seasonal differences (*p* > 0.05) being observed for conductivity, whilst for sites M1 vs M4 (*p* = 0.035) and M1 vs M5 (*p* = 0.031) for conductivity, M1 vs M5 and M1 vs M4 for water temperature and M2 vs M5 (*p* = 0.006) for TDS. Sediment organic matter (SOM) content generally increased from site M1 to M3, showing a decreasing trend across all three seasons (Fig. 2d). Thus, significant SOM content variation (F = 8.24, *p* = 0.011) was observed across seasons, but no significant differences (F = 0.98, *p* = 0.435) were observed for sites. Highest and lowest SOM contents were observed during the cool–dry and hot–wet season, respectively, due to varying water flows (Fig. 2d). Pairwise comparisons identified significant differences between the cool–dry and hot–wet seasons (*p* = 0.016).

### Microplastic distribution

**Fig. 3:**
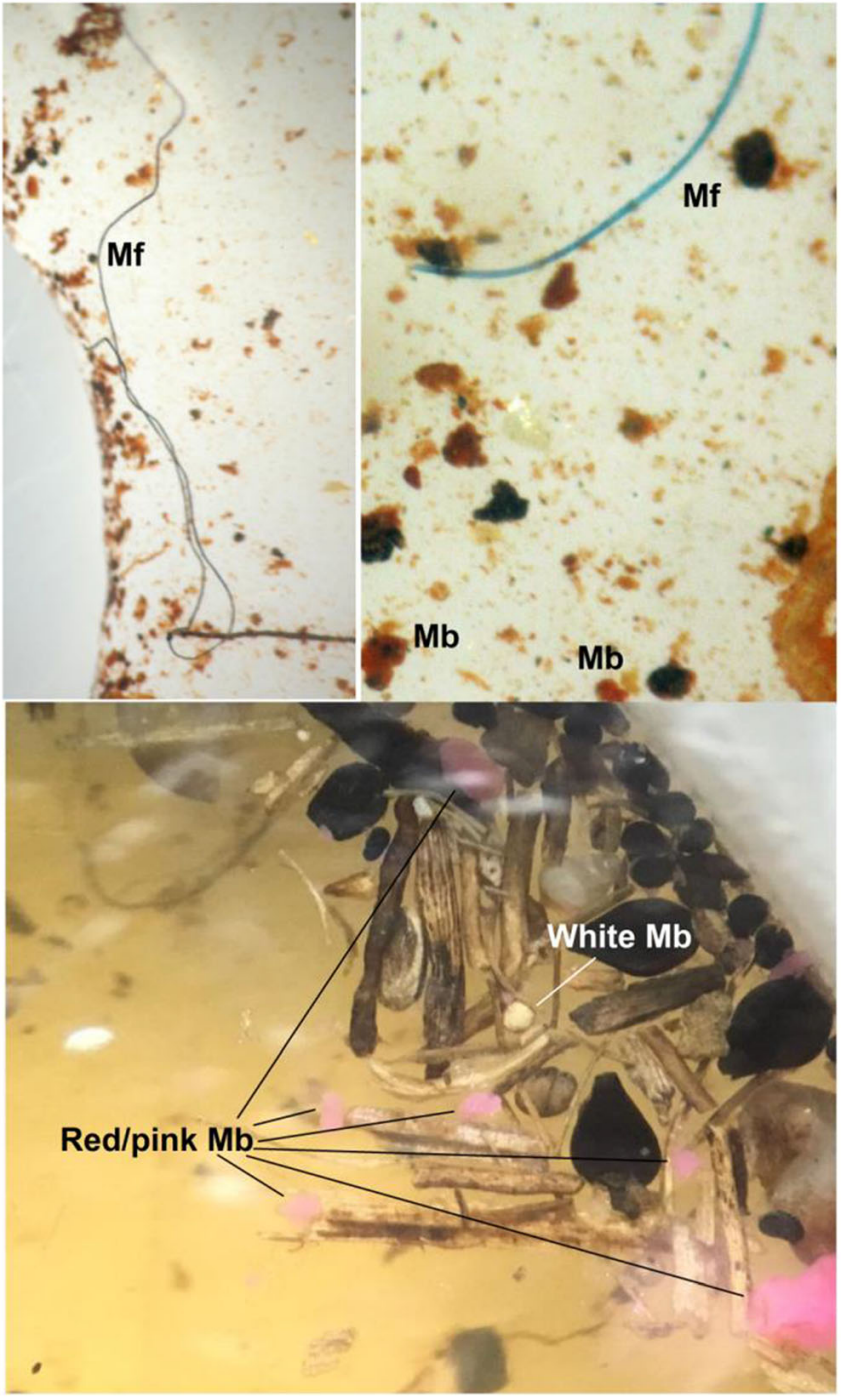
Examples of microplastic (i.e. microfibres (Mf); microbeads (Mb)) that were observed in the sediment samples, mixed with organic matter

**Fig. 4:**
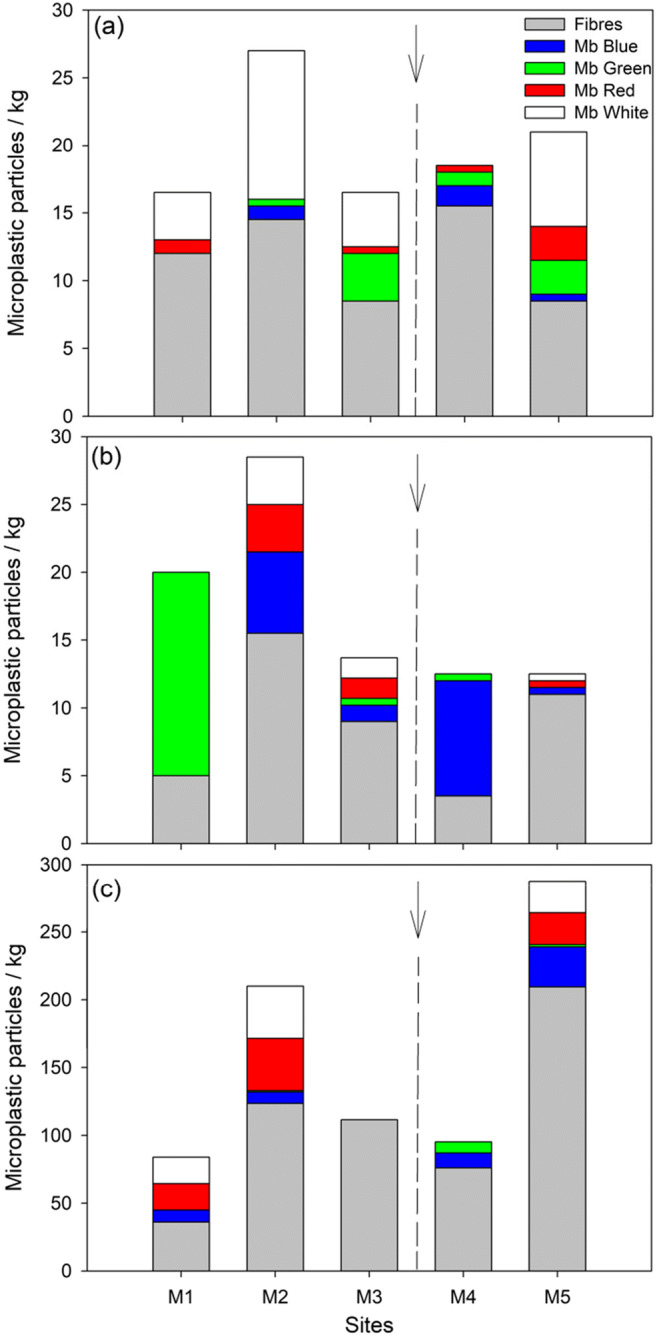
Mean numbers of microplastic particles kg^−1^ sediment along five sites of Mvudi River during (a) cool–dry, (b) hot–dry and (c) hot–wet seasons. Note differences in y–axes scaling. Mb, microbeads. The arrows represent the position of sewage treatment works

Two thousand four hundred six microplastic particles (cool–dry—199 items; hot–dry—217 items; hot–wet—1990 items) were collected during the study, which were mostly fibres and microbeads (i.e. blue, green, red, white) (Figs. 3 and 4). The mean microplastic densities were generally low during the cool–dry (mean range 16.5 ± 4.5–27.0 ± 5.0 microplastic kg^−1^) and hot–dry (mean range 13.0 ± 4.0–29.0 ± 10.0 microplastic kg^−1^) seasons, and highest during the hot–wet (mean range 76.0 ± 10.0–285.5 ± 44.5 microplastic kg^−1^) season. Accordingly, we reject the null hypothesis of equal concentrations among seasons. Fibres dominated the microplastic abundances, followed by white microbeads (Fig. 4). The effect of the wastewater sewage works (i.e. M4) was, however, not obvious due to the high presence of raw sewage along the entire river system, either in terms of type or density. Therefore, we accept the null hypotheses of similar concentrations and types among sites. However, at the most downstream site (M5), abundances were particularly marked in the hot–wet season.

**Fig. 5:**
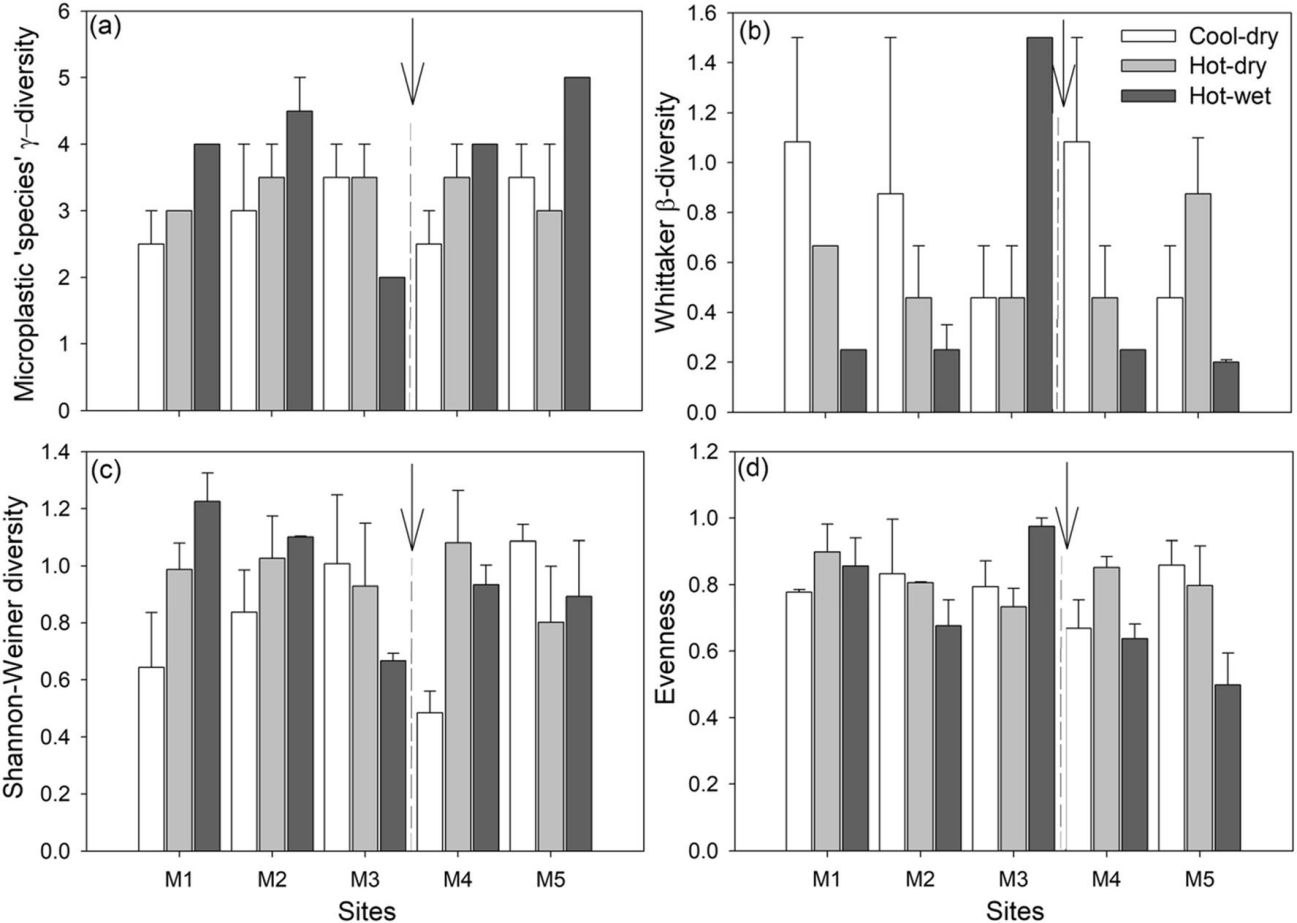
Mean (±standard error) (a) microplastic particle ‘species’ (i.e. γ-diversity), (b) Whittaker β-diversity, (c) Shannon–Wiener index and (d) evenness across study sites and seasons. The arrows represent the position of sewage treatment works

The microplastic particle/colour types and ‘species’ (i.e. γ-diversity value) varied across seasons, with high amounts of microplastic particle ‘species’ being observed mostly during the hot–wet season (Figs. 4 and 5a). The γ-diversity values ranged between 2 and 5, with the Whittaker β-diversity values ranging between 0.20 and 1.50. Whittaker β-diversity generally decreased from the cool–dry to hot–wet season, excepting M3, with low γ-diversity sites having high Whittaker β-diversity values and vice versa (Fig. 5). The Shannon–Wiener diversity index and evenness did not show clear pattern across seasons or sites (Fig. 5c–d). Significant differences were observed for taxa richness across seasons (F = 3.94, *p* = 0.042) and similarities (*p* > 0.05) for the other indices. Tukey’s pairwise comparisons indicated significances differences (*p* = 0.037) between the cool–dry and hot–wet seasons. No significant differences (*p* > 0.05) were observed for all indices across sites.

No significant relationships (*p* > 0.05) were observed for the different environmental variables (i.e. conductivity (*r* = − 0.05), TDS (*r* = − 0.15), pH (*r* = 0.39), temperature, SOM (*r* = 0.18)) and total microplastic particle abundances across the different seasons. Accordingly, we accept the null hypothesis of a lack of effect of abiotic variables on microplastic concentration.

## Discussion

This study investigated the effects of urban development, and in particular wastewater treatment works, on the distribution, type and occurrence of microplastics along a subtropical river system. Overall, we found that microplastic distribution and type generally varied along the river system, with the impact of the wastewater treatment plant not apparent. Furthermore, the microplastic concentration was highest during the hot–wet season, with abundances generally similar among sites. Whilst certain abiotic variables differed significantly across sites and seasons, we also observed no significant correlations between microplastic pollution loads and measured environmental variables (i.e. conductivity, TDS, pH, temperature, SOM).

Based on these results, we thus reject our first and second hypotheses as microplastic concentrations were not increased by the wastewater treatment plant. Further, microplastic types were dominated by both microfibres and microbeads across all sites, but in some cases were lower downstream, suggesting that the wastewater treatment plant was playing some role in removing microplastic, or influencing loadings via dilution effects (due to the pumping-in of wastewater). However, during the cool–dry and hot–dry seasons, site M2 exhibited marked concentrations, with washing and bathing areas under a bridge as well as a university and settlements that contribute sewage directly into Mvudi River in this area. Thus, the microfibre notion is supported in that wastewater is a major contributor, and these microfibres occur where there is a lot of sewage (Habib et al. 1996; Zubris and Richards 2005; Browne et al. 2011; Henry et al. 2019; Liu et al. 2019). Microfibres are found in clothing, and when these clothes are washed, it has been estimated that, for example, a synthetic jacket can release 1.7 g of microfibres (Hartline et al. 2016). In the end, about 40% of these fibres are released into the aquatic environment where they contribute to plastic pollution, with microfibres now estimated to comprise >80% of total microplastics within aquatic ecosystems (Browne et al. 2011; Hartline et al. 2016). This suggests that these microfibres are mainly derived from sewage (Henry et al. 2019; Liu et al. 2019), which comes from the domestic households found within the study area. Thus, the study provides insights into the effects of urbanisation on microplastic abundances along a subtropical river system.

In addition, in relation to our third hypothesis, the significant increase in microplastic concentrations during the hot–wet season likely emanates from increased discharge into the Mvudi River that moved microplastics to the sampled downstream sediments, as well as additional sewage leaks that occur in that season. Therefore, our results show that seasonal context drives differences in the microplastic concentration, with the hot-wet season associated with the highest pollution loads. Previous works have similarly displayed seasonal variations, with river sediments acting as both sinks and sources of microplastics (Nel et al. 2018). That same study found that the winter season was associated with elevated microplastic concentrations due to reductions in flow rates and increased sediment deposition. Accordingly, there is a strong relationship between seasonality, suspension and deposition, with dry periods characterised by high rates of deposition where river sediments act as a sink, whilst wet periods are associated with resuspension of microplastics whereby sediments become a source. This also corroborates the differences in SOM in the present study among seasons, with SOM being highest in the dry seasons. However, results of Nel et al. (2018) additionally contrast those in the present study, where sediment microplastic concentrations were greatest in the hot–wet season, indicating that other, site-specific factors may govern microplastic concentrations at local scales among seasons.

The high microplastic concentrations observed were principally from sites located upstream of the wastewater treatment plant, indicating the presence of other significant sources of microplastics to the river, which wastewater treatment may reduce (Dris et al. 2015). These sources and activities include informal and formal human settlements, water abstraction, riparian brick making, washing and bathing, subsistence and commercial agriculture, sewage discharge/spillage and solid waste disposal/dumping. As such, all the sites which were near the town and downstream of wastewater treatment works showed high variability in microplastic abundances. The present study also identified consistencies in microplastic type, with microfibre concentrations high throughout the sampling sites compared to microbeads, similar to several studies such as Hoellein et al. (2014), Henry et al. (2019) and Liu et al. (2019). It is important to highlight that wastewater treatment plants receive large amounts of microplastic, particularly microfibres, from washing clothes, whereas microbeads are mostly from cosmetics, personal care and cleaning products from the surrounding residential areas. Whilst most microplastics are removed during wastewater treatment, a significant proportion is released into the local aquatic environment. Due to the high capital costs of wastewater treatment plants, upgrading wastewater treatment operations is not a feasible solution to microplastic pollution in the short to medium term in much of the developing world, including the current study area. Thus, it has been reported that whilst some wastewater treatment plants can remove microbeads at an efficiency of >90%, other types cannot be removed effectively, leading to types such as microfibres causing significant environmental pollution (Carr et al. 2016; Murphy et al. 2016). The lower microbead concentration across sites in our study could also be explained by the density separation method employed, which could have inadequately removed microbeads from the sediments (Nel et al. 2018). However, we found high recovery rates overall.

Across all of the seasons, there was no clear difference in microplastics type; however, densities and diversities tended to be highest in the hot–wet season as most of the microplastics are quickly washed downstream and are unable to settle and degrade, making colours easily identifiable. According to Mani et al. (2015), this implies that sources of microplastics in river catchments are diverse and may vary within and across the catchment. As such, there are various external factors governing the particle distribution which may account for greater concentrations upstream of the wastewater treatment, such as river flow, water depth, substrate type and plastic characteristics (Castañeda et al. 2014; Eerkes-Medrano et al. 2015; Klein et al. 2015). On the other hand, despite abiotic variables differing significantly in certain instances across sites and seasons, the present study did not find any of those parameters to be significant predictors of microplastic loads across sites, rejecting our fourth hypothesis. Overall, the present study found marked seasonalities in microplastic concentrations in an understudied geographic region, whilst types and concentrations among sites were more similar. Further work is required to elucidate the effects of wastewater treatment works on microplastic characteristics in the Global South.

## Supplementary Information

ESM 1 (XLSX 10 kb)

## Data Availability

All data generated or analysed during this study are included in this published article (and its supplementary information files).
